# Tetramine poisoning in China: changes over a decade viewed through the media’s eye

**DOI:** 10.1186/1471-2458-14-842

**Published:** 2014-08-13

**Authors:** Yi Li, Yanxia Gao, Xuezhong Yu, Jingmin Peng, Fei Ma, Lewis Nelson

**Affiliations:** Emergency Department, Peking Union Medical College Hospital, Chinese Academy of Medical Sciences, Beijing, 100730 P.R. China; Emergency Department, The First Affiliated Hospital of Zhengzhou University, Zhengzhou, 450052 P.R. China; Medical Intensive Care Unit, Peking Union Medical College Hospital, Chinese Academy of Medical Sciences, Beijing, 100730 P.R. China; Department of Emergency Medicine, New York University School of Medicine, 455 First Avenue, Room 123, New York, New York 10016 USA

**Keywords:** Tetramine poisoning, Poisoning control, China

## Abstract

**Background:**

Tetramine, or tetramethylenedisulfotetramine, is an internationally banned compound that had been used primarily as a rodenticide. Despite its regulatory status, there are widespread reports of its intentional use in human poisonings, primarily in China, and often in mass poisonings. Enhanced governmental regulations were implemented in 2003 to further reduce the availability of tetramine, though the effects of these regulations, and the current use of tetramine, remains unknown.

**Methods:**

Reports from the website of the China News Agency were collected from 2000 to 2012. Details such as the location, date, and intent of the events were compared before and after the regulations were implemented.

**Results:**

There were a total of 148 events during the study period (95 from 2000 to 2003, and 53 after 2003). There were a total of 3526 victims, including 225 fatalities. The majority of the events were homicidal/terroristic in nature. The incidence of events fell after 2006. More poisoning events occurred in central China, such as Henan and Jiangsu province, and an increase was noted in April and September.

**Conclusion:**

Tetramine poisoning events, as reported in the national Chinese media, fell after the implementation of strict regulation on tetramine. The causal relationship is not known.

## Background

Tetramine (tetramethylenedisulfotetramine) is a highly toxic compound that was previously used as a rodenticide. This use has been widely discontinued across the globe due to the risk and severity of human exposures. Tetramine is on the World Health Organization’s list of “extremely hazardous” pesticides, and it is believed that as little as 10mg can be lethal to an adult [1]. This is significantly more toxic than the other currently used rodenticides, such as long-acting anticoagulants, strychnine, fluoroacetamide, and zinc phosphide [2]. As a “cage convulsant” tetramine exerts its toxicity through blockade of the chloride channel on the neuronal gamma-amino butyric acid (GABA) receptor complex. This results in neuronal depolarization and typically produces signs of neuroexcitation, including dizziness, paresthesia, and vomiting, and the effects may progress to seizures and status epilepticus in patients with more severe toxicity. Given its extreme toxicity and persistent availability despite being banned, many remain concerned about its public health implications and the potential for use as an agent of terrorism. Although definitive data do not exist, tetramine appears to be among the chemical agents most widely used for homicide in China. For this reason, the media coverage of tetramine poisoning in China has been intense and the consequences of such visibility are potentially concerning [3, 4].

The most consequential use of tetramine as a nefarious poison occurred on September 14, 2002 in Nanjing, Jiangsu province. In this single event, more than 300 persons were poisoned with tetramine by food consumed at a contaminated snack bar. There were 42 deaths, all children [5]. In response to the events, on August 15, 2003, 9 ministries, including those for agriculture and health, as well as the police, crafted regulations that strictly controlled the availability of tetramine and forbid its rodenticide use [6]. In May 2013, in Hebei province, China, two children in kindergarten were poisoned with tetramine by the head of a rival school, and this event gained public attention around the world [7].

We were interested in evaluating the media coverage of tetramine poisoning within China to gain a better understanding of the epidemiology of such poisonings, such as the regional variation, demographics of both perpetrators and victims, and the effect of governmental controls on the availability of tetramine implemented in 2003. We hoped to assess the impact of enhanced regulation on the availability of this dangerous, and largely banned, substance.

## Methods

The website of the China News Agency (CNA) was searched using the term “tetramine” inclusive of the years 2000 (the date of inception of CNA) to 2012 [8]. This news agency has served as a large national information resource in China since its foundation. News reports were independently reviewed by two authors (YG and FM), both of whom are physicians, and the province, place, numbers of persons affected, clinical outcomes (such as death), year, month, and manner/intent of the poisoning event were recorded. The data were entered into an excel spreadsheet. An attempt was made by the reviewers to identify duplicative reports based on the timing, location, and victim counts. Comparisons were made among several variables between years, specifically focusing on changes that followed the regulations implemented in 2003. To assess the completeness of coverage of the CNA, the Xinhua News agency was searched for online news reports regarding tetramine for the years 2002–2004. The Ethics Department of the Peking Union Medical College Hospital waived review of this study because it was a non-interventional, retrospective review based on media reports that do not provide identifying patient data.

## Results

**Figure 1:**
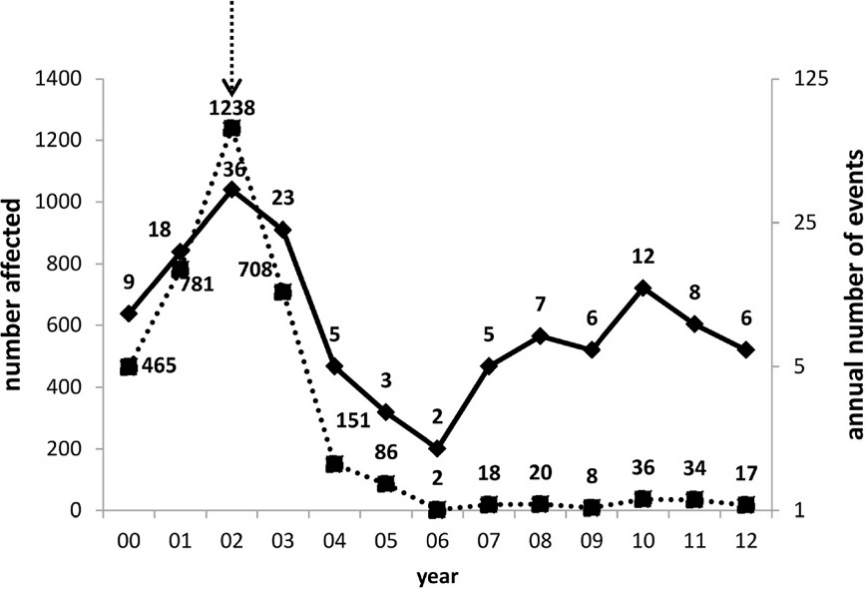
**Annual number of events (right axis, solid line) and number affected (left axis, dotted line).** Number of events and number affected by year. The solid line is number of events per year, the dotted line is the poisoning number.

**Figure 2:**
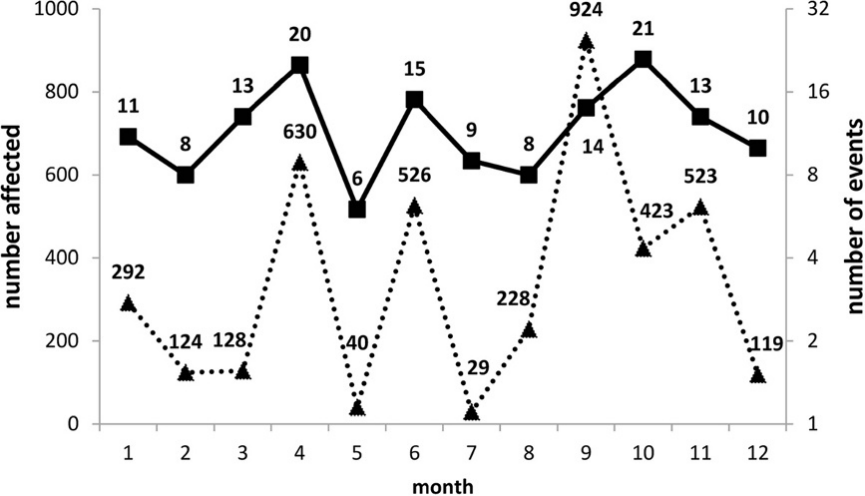
**Number of events (right axis, solid line) and number affected (left axis, dotted line) by month average over 13 year (2000–2012).** The x-coordinate is month.

**Figure 3:**
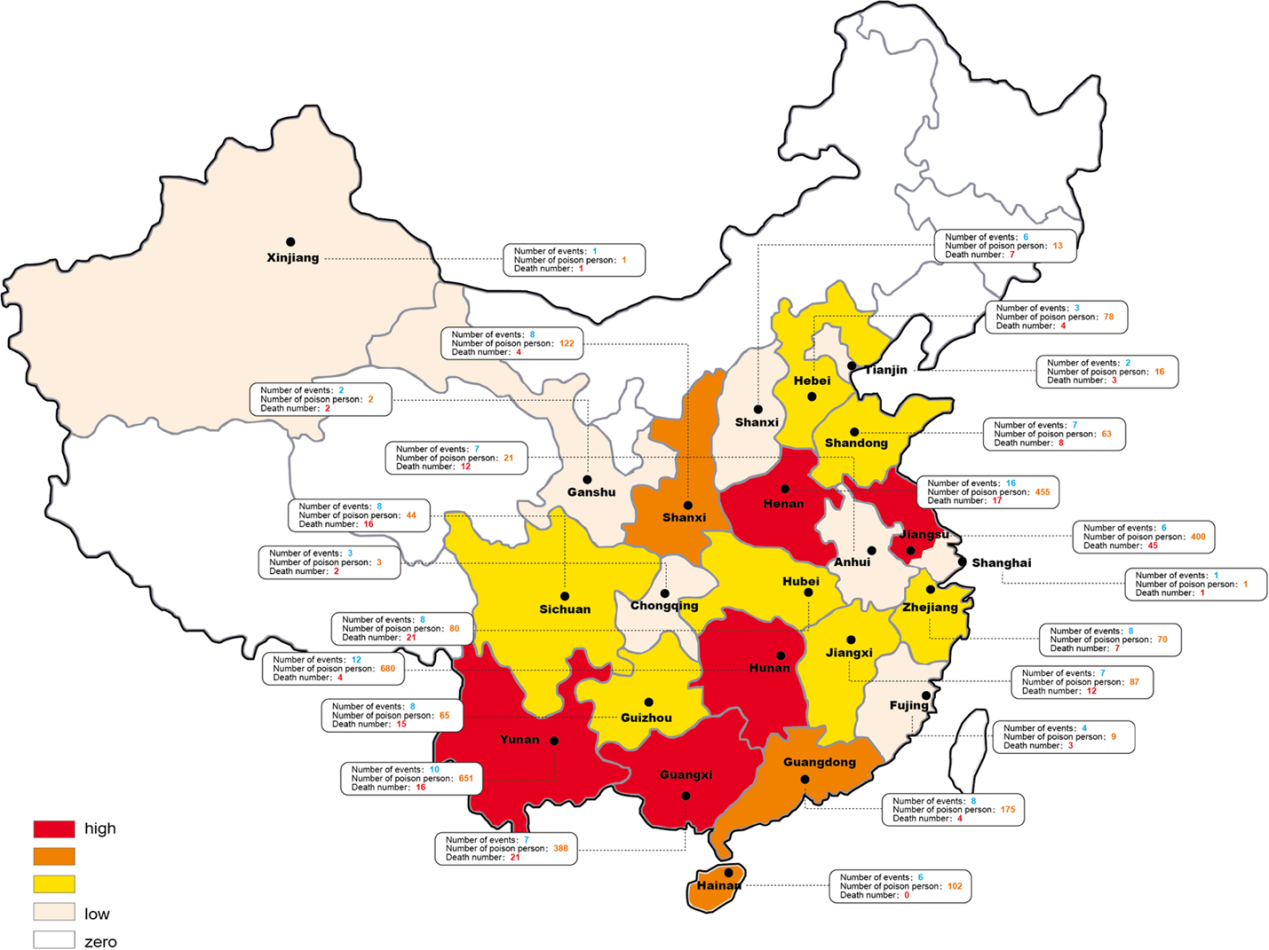
**The distribution of tetramine events.** The colors on the map darken as the number of events increase.

A total of 148 events involving exposure to tetramine was reported; 95 occurred before 2003, and 53 after. A total of 3526 persons were affected, including 225 deaths. All of the reported events involved the ingestion of the toxin. The typical reported clinical findings were nausea, vomiting, dizziness, seizure, or coma. The peak in both the number of poisoning events and the number of victims occurred in 2002. When the enhanced regulatory efforts were implemented in 2003, the number of events dropped precipitously and remained low from 2006 through 2012 (Figure 1. Arrow downwards show the year regulation executed). The number of reported individual poisoning events has risen slowly in the past several years, although the number of affected patients remains small [7]. There are more reports of tetramine poisoning generated in April and September (Figure 2). Details of the poisoning events based on the individual province are depicted in Figure 3.

Henan was the province with the greatest number of events, followed by Hunan, and Yunan, in order. Since 2004, the provinces that have the most victims are Henan and Hunan. Events with the largest number of victims affected occurred in 2002 to 2003, and were in canteen or restaurants, were homicidal in nature, and resulted in more than three hundred victims. The event with the greatest number of fatalities was enacted by a vendor in a restaurant of Jiangsu province in year 2002, and affected 395 persons including 42 deaths. Overall, there were 26 events with more than 30 victims (involving 2111 persons and resulting in 75 deaths); all occurred prior to 2003 [7].

The specific locations for the event are listed in Table 1. Restaurants were the site of poisoning for the greatest number of victims. Homicide was the most common intent of the poisoning, and farmers, restaurant staff, and students the most common perpetrators. The specified intent of the poisoning event and the occupation of the perpetrator are depicted in Tables 2 and 3, respectively.

**Table 1:** **Details of poisoning based on the location of the event**

Location of event	Number of events	Number affected	Manner			
			Unknown	Unintentional	Homicide	Suicide
Home	91(61.49%)	360(10.21%)	7(4.73%)	7(4.73%)	69(46.62%)	8(5.41%)
Public place	54(36.49%)	3162(89.68%)	15(10.14%)	9(6.08%)	30(20.27%)	0
-Restaurant	44(29.73%)	3104(88.03%)	9(6.08%)	1(0.68%)	34(22.97%)	0
-Company/school/construction site	10(6.76%)	58(1.65%)	6(4.05%)	1(0.68%)	3(2.03%)	0
Unknown	3(2.02%)	4(0.11%)	0	1(0.68%)	2(1.35%)	0
Total	148(100%)	3526(100%)				

(Number affected (percentage)).

**Table 2:** **Details of the events based on the manner of poisoning**

Manner of poisoning	Number of events	Number affected	Number dead
Homicide	106(71.62%)	2918(82.76%)	177(78.67%)
−3 affected per event	36(24.32%)	328(9.30%)	49(21.78%)
−3-30 affected per event	44(29.73%)	479(13.58%)	53(23.56%)
− ≥ 30 affected per event	26(17.57%)	2111(59.87%)	75(33.33%)
Unintentional	11(7.43%)	78(2.21%)	7(3.11%)
Suicide	8(5.41%)	10(0.28%)	8(3.56%)
Unknown	23(15.54%)	520(14.75%)	23(10.22%)
Total	148(100%)	3526(100%)	225(100%)

(Number affected (percentage)).

**Table 3:** **Occupation of the perpetrator (% of number of events)**

Group	Number of events	Number affected	Intent			
			Unknown	Unintentional	Homicide	Suicide
Agriculture	47(31.76%)	441(12.51%)		2(15.38%)	43(43%)	2(16.67%)
-Farmer	47(31.76%)	441(12.51%)		2(15.38%)	43(43%)	2(16.67%)
Food service	25(16.89%)	1695(48.07%)			23(23%)	2(16.67%)
-Restaurant staff	17(11.49%)	1670(47.36%)			17(17%)	
-Commerce and service trade^1^	8(5.40%)	25(0.71%)			6(6%)	2(16.67%)
Other occupations	29(19.59%)	278(7.88%)			29(29%)	
-Student	10(6.76%)	542(15.37%)			10(10%)	
-Migrant laborer^2^	4(2.70%)	4(0.11%)			4(4%)	
-Doctor	3(2.03%)	78(2.21%)			3(3%)	
-Teacher	3(2.03%)	136(3.86%)			3(3%)	
-Unemployed	3(2.03%)	23(0.65%)			3(3%)	
-Cadre^3^	2(1.35%)	3(0.09%)			2(2%)	
-Worker^4^	2(1.35%)	2(0.06%)			2(2%)	
-Retired	1(0.68%)	23(0.65%)			1(1%)	
-Others	1(0.68%)	9(0.26%)			1(1%)	
Unknown	47(31.76%)	570(16.17%)	23(100%)	11(84.62%)	5(5%)	8(66.66%)
Total	148(100%)	3526(100%)	23(100%)	13(100%)	100(100%)	12(100%)

^1^Persons involved the manufacture and transportation of the food.

^2^Farmers who leave the village for jobs in the cities.

^3^Person who works for the government.

^4^Factory worker.

Search of the Xinhua News Agency turned up no additional reports of tetramine poisoning during the three year search period.

## Discussion

Tetramine is an odorless, tasteless, water-soluble toxin, which may be surreptitiously administered to unsuspecting victims (homicide), used in suicide, or cause secondary poisoning through contamination of food when applied nearby as a pesticide (unintentional). Typically, patients rapidly develop convulsions, coma, and other neurological disorders. The seizures are difficult to terminate and often lead to death or irreversible central nervous system harm. Tetramine poisoning was first reported in the United States in May 2002 preceding the Nanjing, China event by only several weeks. In this case, a child inadvertently ingested a rodenticide that was imported from China and was purchased in a local New York City grocery [5]. Since these events, its use in homicide and its potential for use in terrorism, have garnered international attention [5].

In China, regulations were implemented in 2003 to address the safety risks associated with tetramine and other lethal rodenticides. This included the cooperation of nine national ministries (Ministries of Agriculture and Health, The National Development and Reform Commission Committee, The State Administration for Industry and Commerce, National Quality Supervision and Inspection, Inspection and Quarantine, State Environmental Protection Administration of China, The State Food and Drug Supervision and Management Bureau, and State Administration of Work Safety ) and law enforcement, which worked as a special team in collaboration with the local government [6]. The agencies reviewed all aspects of tetramine leading to its use in poisoning, including the production, sale, use in agriculture and other circumstances, storage and environment contamination control, emergency disposal of the events, treatment of the patients, and criminal punishment. Inspections for the sale in markets as well as for illicit production were performed in collaboration with the local governments.

Obvious decreases before and after 2003 are noted for most tetramine affected provinces [14 in 23 provinces]. Five provinces (Anhui, Hunan, Henan, Yunan, and Zhejiang) had more than 5 events after 2003. Hebei and Zhejiang had more events after 2003 than before (0 versus 3, and 3 versus 5 respectively). It is not clear what accounts for the differences, although the small numbers may simply represent random events. Interestingly, a report in the Chinese literature shows that all the seven tetramine events in Anhui province in 2005 occurred in the Fuyang [9]. This city is a major rodenticide producer and marketing center in China, and easy availability may account for its greater use.

Events occurred primarily in public places such as restaurants and schools, and were primarily homicidal in nature [Table 1]. There were 26 events involving more than 30 poisoned persons, affecting 2111 persons and resulting in 75 deaths. Farmers, restaurant staff, and students are the top three occupations involved as perpetrators of homicidal poisonings with tetramine. It can be speculated that farmers had access to the poison, restaurants had access to the food, and students were often under stress or prone to impulsive behavior.

The majority of events occurred in central China, such as Henan or Jiangsu province, which are highly agrarian regions. There were no or few events in the Western or the Northeast areas of China. The climate in the northern provinces may limit the need for outdoor rodenticide use in farming, reducing the availability of tetramine for impulsive use. Similarly, the seasonality of poisoning, with the greatest frequency in April and September, may arise from the high rate use of the rodenticide during cultivation or harvest seasons. Similar findings are reported by Xian [10].

The data update and expand a previous study using similar methodology. Using open source data from Xinhua News Agency from 1998 to 2002, Coddy, et al. [11] identified 36 events in 18 provinces (out of 31 in mainland China). Their findings overlapped regionally with ours, despite the difference in time period of evaluation [11].

The most important finding of our research is that the reported use of tetramine for poisoning events decreased following the expanded 2003 regulations. Although we cannot state with certainty why this occurred, it is most likely a result of this public health intervention. It is generally accepted that the primary prevention of any poisoning occurs through the reduction and control of the availability of the toxin. However, it is also possible that the media attention and the seriousness of the harm caused may have reduced the use in poisoning.

To our knowledge, our study is the first to attempt to evaluate the effect of this specific intervention on the public consequences of the availability of tetramine. Other public health efforts have shown similar beneficial results, such as the prevention of pesticide poisoning in Sri Lanka [12] and the Poison Prevention Packaging Act in the US of 1970, and paracetamol pack size limits in the UK [13]. To further reduce the public impact of tetramine poisoning, it may be helpful to focus on the areas with high production of rodenticide, enhanced tetramine surveillance during farming season, provide public education about the lethal threat especially for those having easy access to the toxin, and to adopt strict food safety supervision rules in restaurants and schools. Additionally, detection and management of poisonings may be improved through an enhanced surveillance system involving poison control centers, as occurs in Europe, the United States, and other parts of Asia [14].

### Limitations

Poisoning events are not always identified or reported by the media, limiting the completeness of the data we collected. We have tried to minimize the limitation by comparing the three year data (from year 2002 to 2004) between CNA and Xinhua News agency, and found no extra report from the later. Though there are other news agencies’ websites, they suffer similar limitations. There is no reason to believe that there has been selective reporting of tetramine poisonings nor that reporting was systematically misleading. However, the accuracy of the reports themselves could not be validated. Although we attempted to identify duplicative reports, the variations in reporting style and content may have prevented recognition.

## Conclusion

Tetramine poisoning events are common in China and have resulted in thousands of deaths, primarily homicides, over the past 13 years. There are seasonal and regional differences in the poisoning events. The implementation of intensive regulation in 2003 coincided with a decreased number of media reported events and reported numbers of victims.
